# Gaining resolution when creating imagery of aging

**DOI:** 10.3389/fsoc.2022.957491

**Published:** 2022-12-01

**Authors:** Jacob Sheahan

**Affiliations:** School of Design, RMIT University, Melbourne, VIC, Australia

**Keywords:** design for aging, images of aging, cultural probes, imagery resolution, interaction design

## Abstract

In seeking to support healthy aging, designers have struggled to reduce their assumptions and biases toward older adults, been seen to interpret the worlds of later life through unfiltered imagery, as well as engage with stigmas, ultimately diminishing the technologies they construct. This article seeks to critically analyse this state-of-the-art from a design research perspective while engaging with the growing interdisciplinary study of aging and technologies. Toward this, we proposition “resolution” as a concept indicative of the level of detail that seeks to characterize the fidelity that representations of later life have. This concept is explored through a cultural probe study that investigated the sentiments of several older Australians regarding the inequities and social isolation brought on by the COVID-19 pandemic. Providing a diary alongside photovoice and mapping tasks, the study captured perceptions of social technology, practices, networks, and wellbeing, offering a diverse and complex picture of aging and technology. Through reflexive thematic analyses of some of these materials, this case study offers designers pathways to understanding and including older adults in their work. In determining the resolution of these images of aging, we discuss how transparency about the limitations and qualities of such participatory methods through incorporating reflexivity can influence the degree of detail such imagery gains. Ultimately this concept builds on the notion of participation configuration, supporting designers to realize better images of aging and representations of later life.

## Introduction

When engaging a new brief or in an unknown context, designers will draw on not only their own experiences and research but also the wealth of public information on a given topic. When enquiring about later life, this process often sees intuitive sourcing of publicly available imagery and anecdotes into propositions, ignoring richer ethnographical and collaborative modes with older adults (Peine, 2019). Scholars suggest that in drawing on such data through their iterative process, designers (those involved in industrial, product, service, and interaction practice) will often over-emphasize cognitive and physical decline as matters of concern, with a tendency to form pessimistic social images of aging (Dankl, 2017). The combination of selective sources and concealed processes of determining outcomes can be seen to result in many artifacts that inadvertently continue stereotypes and stigmas of older adults through factors such as a *lack of aesthetic appeal, accentuated social signifiers, poor affordance, and neglect of privacy* (Li et al., 2020). Such elements are endemic across designed technologies for later life (Peek et al., 2016; Garde et al., 2018).

The employment and production of negative imagery is not limited to the design fields, nor is mis-presentation the only issue with how media organizations portray later life, however, the COVID-19 pandemic has been seen to inflate them. Literature reviews on images of aging have documented the underrepresentation of older adults in media, especially women, as well as being characterized with forgetfulness and rigidity (Ylänne, 2012; Bai, 2014). These issues were persistent before the COVID-19 pandemic, with scholars documenting how media imagery during that event has further ostracized this cohort. As analyzed within the Australian context, visual communication researchers Thomson et al. (2022) found news media coverage to be isolating and marginalizing, generic and vague. Their qualitative representation analysis of aged care captured how those in these settings were represented “with a distant, often de-contextualized or trivialized, and non-representative understanding” (p. 16), such as through visual tropes such as aged and wrinkled hands. Gerontologists Allen and Ayalon (2022) found such misrepresentation was not limited to Australia, noting how newspapers enhanced and reinforced the American public's pre-existing negative associations with residential care during this period. While this article does capture imagery and perceptions of aging during this pandemic, it is helpful to note the existing problems with how older adults are portrayed and how this period has amplified it. Imagery of aging is a well-established subject of gerontological, visual communication and media studies, while growing recognition of its interconnection with the work of designers is forming.

The description of this process as part of a broader “co-constitution of aging and technology”, how images of aging are drawn on in design worlds to form technological artifacts, has been recently articulated by Science and Technology Studies (STS) scholars (Peine and Neven, 2020). This article focuses on images of aging as a concept drawn from STS notions of societal and socio-technical imaginaries (Joyce, 2021), to which design scholars have engaged anthropological and speculative research modes. Whereas, STS theory might be seen to engage with more sensitive thinking about power, users and technology (Östlund et al., 2015), designers have continued to value a creative-led *art of inquiry* (Dankl, 2017), seeking what Ingold (2013, p. 7) describes as capturing “the ethnographic richness and complexity of other cultures while simultaneously opening up to radical, speculative inquiry into the potentials of human life”. This tacit ambiguity of design methods has been attributed to flawed technological interventions and applications, where designers often lack participatory methods and ignore their work's ethical ramifications (Peine, 2019).

This research engages with a broader, ongoing discourse around participatory design practices, which emphasizes the configuration of participation, seeking heightened transparency about biases and the roles of designers (Fischer et al., 2021b). Fischer et al. (2021a) consider how participation is a central concern when the design process is seen as a collaborative effort, with participatory design's attention to empowering people “by bringing designers together with other stakeholders” (p. 1). By contextualizing this discussion through aging and technology, they questioned what a “good” configuration of participation is in practice, seeing such “better” forms are part of a continuous iterative process, requiring reflectivity in the work designers. Whether this means, in the context of imagery of aging, trying to produce more authentic and realistic media brings genuine tension to this work. While we see how such accurate imagery can provide insights into the unique worlds of real people living with Dementia (Harvey and Brookes, 2019), we can also see how “alternative” forms, such as Richards et al. (2012) document in “nostalgic/melancholic or humorously carnivalesque (p. 65)” photography can lead to improvement in self-image and older adults' attitudes (Bai, 2014). In building upon Fischer et al. (2021a) call to support good participation, while also addressing the imagery designers draw on and produce, this article explores methods of participation in image production toward the question of “*what characterizes ‘better’ images of aging created by designers?*”. This question is guided by an effort to bring older adults into image production and seek authentic, or perhaps alternative, images of later life.

In interrogating designers' production of imagery of later life, this article frames the insights from a specific cultural probe study conducted with older Australians. Outcomes focus upon both the methodological learnings and the feedback from participants as we seek to understand whether such participant-centered methods provide improved detail and accuracy to the imagery of older adults. Toward this, we first detail the increasingly participatory-centered methods of image creation across the Design and Human-Computer Interaction fields, forming a hypothesis around the “resolution” gained through such configurations of participation. By interrogating the studies' outcomes, both the resulting materials and feedback gained from respondents, we discuss whether the captured imagery is indeed “better”, is greater detail and authenticity gained, and how designers might be able to characterize better the images they form of later life.

## How designers produce imagery of aging

From early in their training to further into a consolidated practice, designers frequently engage in constructive and exploratory activities toward realizing deeper problems, uncovering important social norms, and forming relevant and engaging artifacts. These practices have been seen to reflect a “field” approach to constructive design research, as opposed to what Koskinen et al. (2015) have also conceptualized as “lab” or “showroom” modes of design, borrowing from social science rather than experimental methods. Often resulting in the appropriating of tools and vocabulary from the ethnographic disciplines, as well as forming their own too, designers engage in an array of ways to observe, study and analyse people (Nova, 2015). However, within the context of later life, this fieldwork has been particularly product-centric, in what Dankl (2017) describes as an overemphasis on “cognitive and physical aging as starting points for design for older adults” (p. 31). Dankl calls for better ways of inquiry and forming images of aging, resisting design-induced stigmas that remain a blind spot for both emerging and established designers (Li et al., 2020).

In contending with this issue, Fischer et al. (2021b) have indicated how different “configurations of participation”, as a result of specific participatory methods, can yield very different images and outcomes. As Fischer et al. (2021a) explore in a separate article, this form of participatory design focuses on knowledge transfer between designers and users. This “mutual learning” takes place when conducted with older adults that can “lead to competent participants, close to ‘expert’ users” (Fischer et al., 2021a, p. 2). Two configurations that enable this learning and have remained prominent and relevant to designers are those of codesign and probes, as “field” focused approaches which have been used to provide a voice and draw on the lived experience of older persons in differing ways.

### Codesigning

Indicative of the growth in participatory cultures of designing, the codesign process has become an essential toolbox for product and service practitioners, known for interrogating ethical practices and modes of community engagement (Sumner et al., 2020). In essence, Sanders and Stappers (2008) establish that codesign “refers to the creativity of designers and people not trained in design working together in the design development process” (p. 7). Evidently, the degree of “working together” is a concern that Tsekleves et al. (2020) have been notable in contextualizing with older adults, highlighting how designers may misrepresent or merge codesign with its sibling “co-creation”. Whereas, the former speaks to shared ownership that designers facilitate and that brings users into the team, co-creation captures “collective creativity” that designers translate, which Tsekleves et al. (2020) see as best describing participatory research with people with Dementia. This record illustrates how the interpretation and application of codesign methods can be highly contested, posing unique complexity and barriers to novice designers, such as students (Bødker and Kyng, 2018). In exploring the life worlds of older adults, codesign methods have frequently been employed toward developed technologies for later life, drawing on personal narratives to generate ideas (Ostrowski et al., 2021b) and directing action research toward creating personal avatars for virtual reality use (Baker et al., 2019).

In exploring these two case studies as examples of producing imagery of aging to inform design decisions, we find these scholars describing the success of participation based upon the authenticity and accuracy of imagery formed. However, we pose how they perhaps could benefit from further interrogating how they capture and process the resulting images of aging. In the first case study, Baker et al. (2019) use codesign to develop 3D Avatars for Virtual Reality (VR) applications, focusing on its role in action research to foster active participation. Describing the use of technology probes to engage participants in several co-design workshops, acting as discreet sessions to create avatars to experience social VR, the scholars sought to bridge exploratory and participatory design stages. The resulting avatars' images demonstrated an interest in youthful appearances but also documented the limited facial expression available. Highlighting the level of participation, the scholars described how the older adults were configured as *technology explorers* who “partnered with us to explore how Virtual Reality technology could be harnessed to provide older adults with opportunities to participate in meaningful social activities (p. 231)”.

In a second example, Ostrowski et al. (2021a) designed social robots with twenty-eight older adults in seven codesign workshops involving interviewing, art-based image making, robot hosting and prototyping, and reflective exercises. The resulting imagery, extracted from the research stories of participants, saw the scholars draw on grounded theory to analyse responses, briefly demonstrating how each story was explored “as a unit to value the context and sequence (p. 5)”. Alongside the analysis of responses, Ostrowski et al. developed these vignettes further into recommendations for conceptualizing a robot for safety and care, as well as methods of installation. Both examples detail how participation remains one of the few key characteristics for indicating the validity of the resulting imagery as a lens for comparability between co-design efforts.

### Probing

As an attempt to critique dominant user-research methods through playful, subjective and provocative approaches to conducting user research, the probe methodology formed by Gaver et al. (1999) in the 1990s remains a relevant tool for designers in exploring later life. The dominant use of and discourse around this method still speaks to its successful integration in both Design and Human-Computer Interaction fields, with ongoing examples of implementation with older adults globally (Wherton et al., 2012; Soro et al., 2016; Annea et al., 2018). When provided to older adults to explore topics of technology as well as wellbeing, probes generally consist of a package of survey materials, camera equipment, social diary or similar methods that are sent to respondents (Chien, 2008; Brown et al., 2014; Wherton et al., 2015a; Mikus et al., 2022). The motives for their use generally focus on creating interactions between designers and users, through empathic understanding, by studying respondents' cultural and personal experiences (Mattelmäki, 2005). As Çerçi et al. (2021) document in interviewing several design researchers that employ probes in their work, they note how this method can “humanize participants” by “expanding or pushing the boundaries of a ‘user’ rather than creating an accurate representation of them (p. 11.)”. This notion suggests imagery gained through cultural probes takes a very different form to that of codesign studies, with the autonomy of respondents rather than participation central.

Considering these qualities and characteristics in cultural probe studies, where the researchers often have liberty with how playful or prescriptive material is, we see how effort is placed on being engaging toward forming interesting results. This is evident in the sensitivity (Brown et al., 2014) had in preparing a probe kit for people living with Dementia, that their outcomes were tied to making the materials “inherently participatory as participants are the active data contributors (p. 1.)”. While contributors in this study, participants were not involved in curating the datasets into several design narratives focused on articulating the challenges people living with Dementia can face. Mikus et al. (2022) describe probes as a method of gathering information through self-reporting that requires embracing care ethics and places a value on “compassion, negotiation, collaboration, and partial perspectives”. An example of this ethical practice was drawing on a cohort advisor to ensure the functionality and appropriateness of the package for older adults, potentially reducing stigmas and improving engagement with their materials. Like the previous study, these scholars formed themes around flourishing and biophilia by coding extracted participant comments. These generative and analytical processes are consistent with the literature, as Sanders and Stappers (2014) establish that cultural probes “evoke inspiring responses from individual participants, with designers using the responses at their own discretion (p. 8)”. Both codesign and cultural probes offer additional engagement with stakeholders, however, both require the designer, as facilitator or creator, to support participants in expressing and communicating their lived experiences, enabling desired portrayals.

### Hypothesizing resolution

In reviewing these pre-existing and prominent approaches that designers use to develop images of aging, we establish the importance and value of participation from scholars and older adults. However, we also see inconsistent efforts to evaluate how imagery is captured and processed. In contending with Fischer et al.'s (2021b) configurations of participation and a question of “*what characterizes ‘better’ images of aging created by designers?*” in light of the many images we can form, hypothesis-making offers this article an additional layer of reflexivity. As Bang et al. (2012) suggest, hypothesis-making exists in constructive design research, often implicitly or tacitly. However, by developing a hypothesis during the research foundation, we can make our motivations clear. A growing tool in unpacking theoretical and practical insights, scholars are drawing on hypothesis-making to explore the limits of empathy in design (Heylighen and Dong, 2019) while Sanders and Stappers (2014) indicate the role of prototypes, such as this cultural probe study, in testing a hypothesis.

In reflecting on the methodical elements of creating imagery of aging, the author considers the potential characteristics of image input and output, examining how images are deliberately produced through specific tools and then analyzing and forming insights through the work of designers. Comparing aspects to the broader graphics and photography concept of resolution, we saw parallels with how this determines the quality (detail) of an image and is dependent on the input from a device (scanner or digital camera) and output medium (onto a computer monitor, printed onto paper, etc.). In hypothesizing the concept of resolution in relation to images of aging, we consider this a characteristic that could enable designers to articulate and ultimately form “better” representations of later life.

Within this hypothesis, we consider how “input resolution” can be understood as the vehicle used to collect data—for example, desktop research relies on journal or news articles, utilizing secondary sources. To capture input resolution, we propose being transparent about how we form or collect images, describing them in terms of low to high input resolution, from secondary sources to primary data. Mirroring the tiers of engagement in participatory design literature, we can conceive a spectrum of image production processes. For example, cultural probe studies' provocative and autonomous aspects could provide a higher resolution than less direct and perhaps more so stigmatizing and biased second-hand imagery.

However, input is only half of the equation, as we suggest “output resolution” helps to describe the processing and analysis of captured imagery into design outcomes. From a photography standpoint, the output resolution is the printing or viewing of an image, which depends on the medium to define how it is represented. For example, a printed image is a number of pixels of dots, which may utilize halftone dot patterns to produce the full tone range of an image, leveraging this optical illusion. In the context of design research, this can be seen as a metaphor for how we go about interpreting data sources and incorporating them into resultant design outcomes. Dorst (2006) describes how a design problem or brief designer has to unearth “hard facts”, that designers often draw on imagery to form possible interpretations and solutions. Output resolution could be seen as the connection between the imagery and the design context, as well as the relevance and strength of the criteria for such interventions.

The following case study is an opportunity to interrogate and reflect on this hypothesis of resolution input and output through a cultural probe as a relevant and helpful approach. The process of hypothesis-making, in the context of reflexive analysis, establishes existing perceptions, ideals, and values the author might form before the study. To motivate and frame the research process, we seek to establish whether gaining resolution through increased transparency and consideration of the methods of creation and analysis, as well as personal biases and misconceptions, if this concept can support designers in forming “better” images of aging.

## Methods

This study aimed to investigate older Australians' social networks, social technologies, and wellbeing. To capture imagery of aging produced around these topics, participants were provided with a posted probe kit to complete over 10 days. Their responses to the various measures, tasks, and activities were analyzed thematically. After completing the probe kit and mailing it back to the researchers, participants were offered a semi-structured exit interview to discuss their thoughts on the topics and feedback on the probe, which all participated in (postage delays due to the pandemic hampered efforts to review the completed probe kits with participants). The cultural probe's contents were materials based on specific activity prompts and measures intended to capture various experiences, perceptions, and understanding. Reflecting the variety found in other cultural probe studies, we expected some participants might prefer photovoice tasks over social network drawing exercises and vice versa, resulting in three components (Photovoice Cards, Social Diary, Workbook) and 35 individual tasks. All participants completed the cultural probe remotely, with only one provided additional time to complete the social diary activities. In addition, other data from measures and scales to assess quality of life, attitudes toward technology, and social engagement, were self-administered to help characterize respondents in relation to the broader participant pool. This article focuses on the photovoice activities, social diary, and exit interview, presenting the development process and analyzing the resulting data thematically. Permission to reproduce images for publication has been provided by all participants, with the process of consent further detailed in Section Study procedure.

### Participants

The study was completed by seven older Australians, each a member of the University of the Third Age (U3A), recruited *via* a posting about the cultural probe study in their local chapter's newsletter. As part of a larger research partnership between RMIT University and U3A Network Victoria, Shaping Connections, invitations to express interest in the study were sent to members in the South-east of Melbourne, Victoria, from Kingston, Cranbourne, Frankston, Glen Eira, and Bayside local chapters. Based on a member profile survey conducted by the Victorian Network, we note 75% of members are women, predominately from 71 to 75 (27%) and 66–70 (24%) age groups, with 48% having obtained a degree or higher compared to 24% for the general population, and 31% of survey respondents living alone (Szwed, 2018). Regarding the cultural probe study, the demographics of the member profile reflected participants well, with an average age of 68 years old (M = 76.29, SD = 8.50), respondents were primarily women (6 women, one man), while education and living conditions were not evaluated, all resided in the south-east of Melbourne and were members of one of the local chapters engaged.

As the study focused on technology use, hobbies, social; habits, and preferences for social connection, participants were invited to discuss these aspects. For example, almost all respondents noted a “regular” level of technology use and skill, with only one suggesting they were at a “beginner” level. All owned a mobile phone, with at least three a table or laptop. All completed the TechPH scale (Anderberg et al., 2019) showing a reasonable overall level of interest and appreciation of technology across the respondents, with low anxiety. When discussing their interests and hobbies, many indicated their involvement in classes with their responding U3A chapters, such as Genealogy, Book club, social studies, archaeology, or photography, alongside traveling around Australia or spending time with family. Regarding how this time with others was spent, almost all preferred regular, in-person catch-ups, whether for a meal, coffee, or walking, as long as it was focused on talking and listening. Using the Lubben Social Network Scale (Lubben and Gironda, 2003) to determine their social engagement, respondents indicated similar levels of social engagement with family and friends across the board. The following backgrounds serve to contextualize each participant:

- Sally: With her son moving in with his family during the pandemic, Sally spends her days helping to look after her grandchildren, preparing meals and watching television together. Most mornings, she plays Pokemon Go on a walk before breakfast, alongside hip exercises to reduce her ongoing bursitis.
- Joan: As a retiree who volunteers at the local visitor information center, Joan lives with her husband on the coast. Her days during the study were spent taking daily walks, completing gardening tasks, and supporting friends and family through in-person chats and online calls.
- Olivia: Due to an immune system badly impaired from the Guillain Barre syndrome, Olivia is housebound and requires daily support from her carers. During the study, she noted how much more accessible her interests had become, such as weekly church and football games.
- Emma: A retiree at 66, Emma enjoys working part-time as a local golf clubhouse gardener and lives in a share house. While looking after her garden and spending time with friends; she also writes short children's books and helps look after neighbors' children.
- Mary: Continuing to work into her late seventies, Mary has set up a home office during the lockdowns but prefers to go into the office to see the other accountants in her unit. At home, with her husband, she enjoys learning and using new devices while also regularly walking their puppy.
- Claire: With her partners separated from her in Canada during the Australian lockdowns, she keeps in contact with him and her family primarily online and in calls. As an avid traveler, she spent her days learning Spanish while preparing to sell her house in Australia.
- Robert: Previously an IT worker, Robert remains engaged with technology through his passion for cameras, though he also provides technical support at his U3A. Outside this, he enjoys spending his time following creative pursuits such as photography and painting, being with his partner and like-minded individuals that join the photography class he teaches.

### Materials

**Figure 1:**
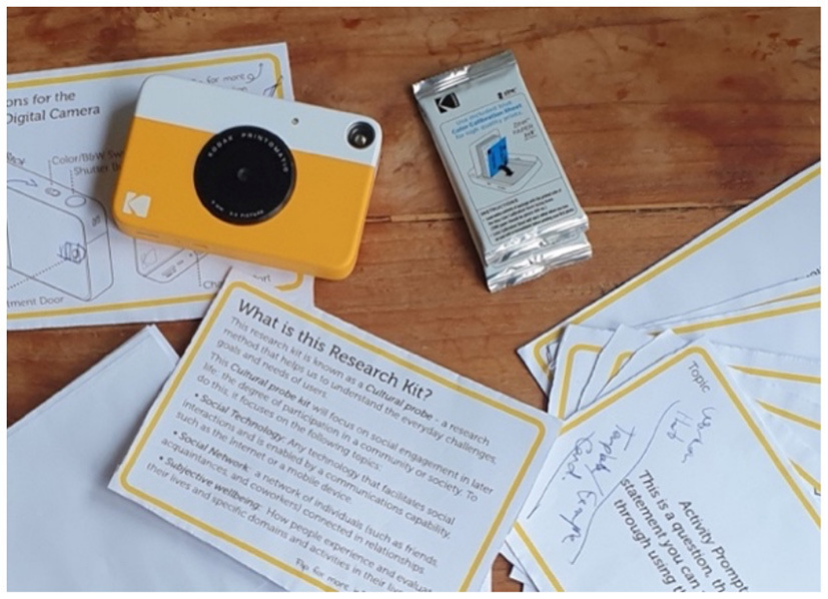
Cultural probe kit and materials.

The cultural probe kit contained three main components—a social activity diary, an accompanying workbook, and a collection of photovoice task prompts with an instant-digital camera—alongside supporting documentation and instructions (Figure 1). Intended for remote and self-sufficient use by participants, all components were printed and sent to participants. In addition, the kit included necessary stationery such as pens and pencils, spare photo papers for the instant-digital camera, and a charging cable in case the camera did not last the week. The optional exit interview was conducted *via* online conferencing software, such as Zoom, or *via* a phone call, per participants' requests. As this article discusses the analysis of the photovoice responses, social diary entries, and exit interview transcript, the development and resultant materials from these elements will be discussed. It is also important to note that as part of the development process, the probe kit was iterated multiple times and tested with a volunteer U3A officer. Reflecting the approach of Mikus et al. (2022), this independent project advisor was consulted to ensure the kit's components, terminology, and distribution were appropriate, potentially increasing engagement and connection. In particular, this involved making changes to language and the text size and replacing activities foreseen not to resonate with this audience, with the tester refining the probe into a more accessible, intuitive, and helpful format.

#### Photovoice tasks

Developed initially by health promotion researchers (Wang and Burris, 1997), Photovoice is a technique that involves placing cameras into participants' hands to help them record, reflect upon, and communicate issues of concern (Budig et al., 2018). Serving as a powerful medium for capturing everyday life, the photovoice component of the cultural probe kit prompted participants to consider and reflect on Social Technology, Social Networks, Wellbeing, and Social Practices. The camera provided to participants, a Kodak Printomatic Instant Print Camera, was intended to provide a novel and interactive platform for capturing imagery, chosen due to its ability to automatically print photos with point-and-shoot ease, as well as record them digitally *via* an SD card. The cue cards were developed to support participants in considering the context of the study—the ongoing lockdowns in Victoria and social interactivity—posing questions and examples that sought to resonate with the situations facing respondents. Each topic responded to their established concepts, such as social technology that captures any technology that facilitates social interaction and has some form of communication capability. This saw a participant respond to a prompting statement (i.e., “COVID-19 has changed our experience and reliance on technologies to keep social”) and question (i.e., “Find and capture examples of technologies you use to socialize around you”), with additional prompting statements for the images taken (i.e., “You might use a device to play games or talk with others…”, or “Perhaps you make use of assistive equipment to access the community…”). Meanwhile, a social network can best be understood as a network of individuals (such as friends, acquaintances, and co-workers) connected in relationships. Participants were prompted with cue card questions (i.e., What do you do with others? Try to capture hobbies, groups or communities that you engage with) that focused on both common interests (i.e., Maybe you have a common interest with others, a hobby or sport, that you do with others?) and online connectivity prompts (i.e., Do you have a digital, online place you talk or meet with others, a regular Zoom call?).

**Figure 2:**
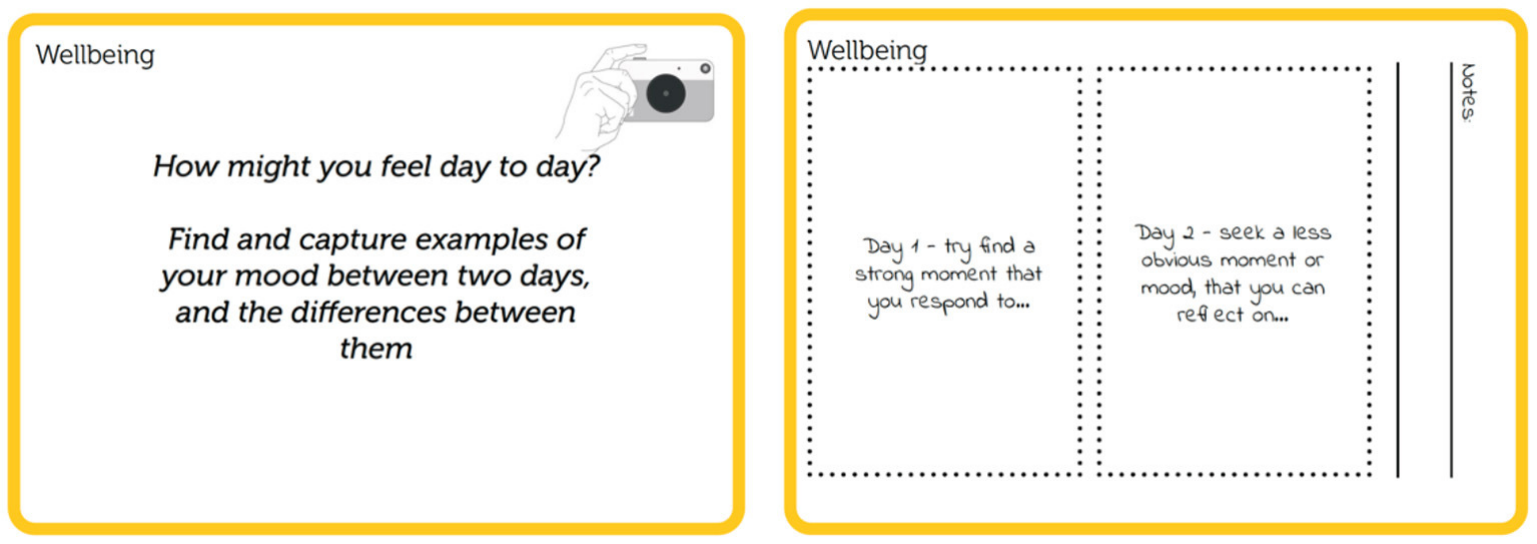
Photovoice prompt cards.

Alongside these, the topic of social practices centered on how everyday actions are typically and habitually performed and are meaningful parts of everyday life. This saw typical photovoice questions focus on representation (i.e., How do you represent yourself to others? Find and capture examples of your social identity, what makes it up, or what you like others to see in you) and sources of support. The final topic of wellbeing not only focused on happiness and life satisfaction (i.e., What might make you happy or proud?) but also on mood change across days (Figure 2). These cue cards were printed double-sided for participants to review the prompts and capture their photographic and written responses, forming a collection of eight activities.

#### Social diary

**Figure 3:**
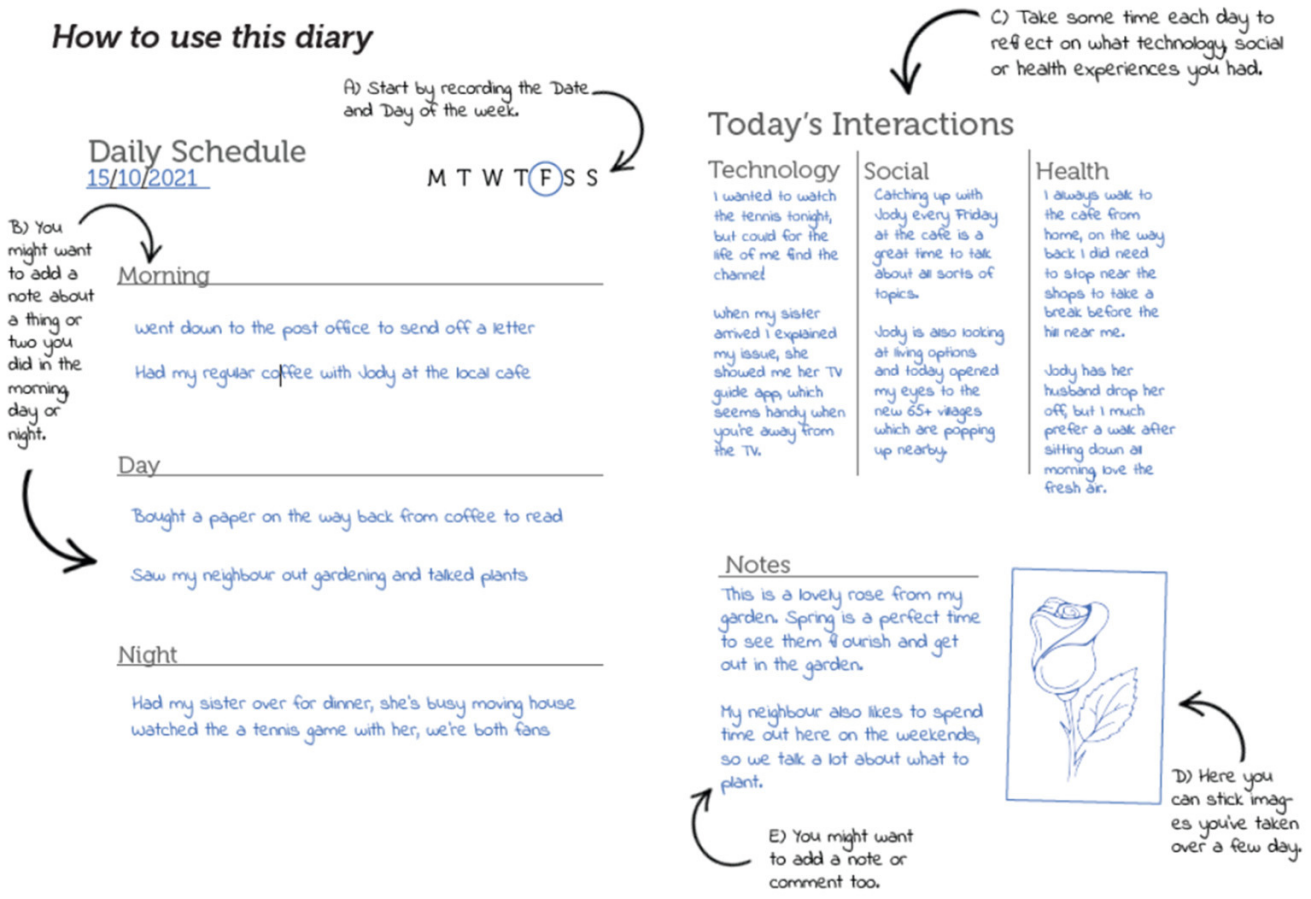
Social diary—daily schedule guide.

Diaries studies form a frequent component and common item in a probe kit, often as a blank book to be filled with observations or a templated format to guide input (Wherton et al., 2012; Thoring et al., 2013). Here, the diary provides a template for recording day-to-to events, relevant imagery, and reflections on daily interactions: with technology, social activities, and wellbeing-related events. Intended to capture actions, events, and reflections over a 7-day week, the diary formed around a “Daily Schedule” template, with simple instructions to follow, allowing participants to record what they wanted (Figure 3). As a way of capturing specific events or determining routine tasks, the diary provides information that might otherwise be absent or forgotten in an interview setting. The additional “Today's Interactions” elements provided a separate section for reflections and review on the day, breaking away from the chronological schedule element to focus on discreet technological, social, and wellbeing experiences.

#### Semi-structured exit interview guide

After completing the cultural probe, participants were invited to complete a semi-structured, post-survey interview. As an opportunity to provide feedback on the cultural probe experience and further explore the study's themes, these interviews varied from 30 min to 1.5 h in length. An interview guide was provided before participants to prepare participants and structure the interviews. It focused first on general and component-specific feedback, then discussed how they understood and perceived social technology, networks, practices, and their wellbeing (Table 1). Alongside addressing these topics, participants were also asked to clarify any images, wording or response they wished to have removed from the data set. These discussions were all conducted *via* the Zoom video conferencing platform, except one *via* a phone call, and were recorded and transcribed for use in the later analysis.

**Table 1:** Semi-structured exit interview guide.

**Topic**	**Questions**
Cultural probe	1. Did you have any issues or complications with using the cultural probe kit? 2. Were you able to complete all activities? 3. Did the Instructions make sense or were they confusing/complicated? 4. Using the camera, could you capture and print off images? 5. How did you find the camera activities and prompts? 6. In the diary, were you able to complete a daily summary and reflections? 7. In the workbook, were you able to undertake the exercises?
Social technology	1. What comes to mind when you consider social technology? 2. Were you able to capture these technologies in your life? 3. Did you have an opportunity to reflect on your actions or practice? 4. How do you feel about the nature of technologies during the pandemic?
Social networks	1. Are you able to think about your individual and wider networks? 2. How has your social network changed over time? 3. Do you find more friends or family, or less in your social bubble?
Wellbeing	1. How do you see your wellbeing? 2. What do you value and find important, or supports your wellbeing?

### Study procedure

Approval for the study was obtained through our institution's human research ethics board, which established that participants' data would be personal and re-identifiable (coded) in nature, before we collected consent from all participants. The study was conducted from August to October 2021, with potential participants required to review the consent information and associated materials before engaging with the study. Once permissions and postage were organized, a parcel containing the study materials was mailed to them. In line with sanitation requirements due to the COVID-19 pandemic, mailed components were handled safely, with all elements sealed or cleaned thoroughly before provision. Participants were given 10 days to complete the kit. Seven days after the kit arrived, participants were sent a reminder to complete the tasks and an invitation to participate in an optional semi-structured interview. Of the seven provided with the mail-out kit, all kits were completed and returned within 2 weeks, and all respondents participated in an exit interview. During the exit interview, participants were invited to review their submitted materials and determine if they wanted anything removed from the data set (i.e., the faces of grandchildren or family members, etc.). This also saw participants provide permission for imagery, written responses, and interview transcripts to be used for publication and dissemination purposes.

### Limitations

In conducting this study, we acknowledge there are limitations and drawbacks to this work. Foremost, we note the small number of participants and single recruitment source—U3A Network Victoria—suggesting that this research might provide a rich and engaging dataset but that the results should not be considered definitive and are reflective of a small sample. Like other cultural probe studies, we also note that these studies often see limited interaction between researchers and participants, which reduces the support and engagement respondents may desire, as an issue only worsened by the conditions of the COVID-19 pandemic and health-related lockdowns (Wherton et al., 2015b; Celikoglu et al., 2017). This saw participants being limited to being able to seek technical support or, in the interviews, to express themselves *via* phone call, email, or videoconference, which did reduce the potential for capturing non-verbal communication and other benefits from face-to-face interactions.

## Analysis

After the probes were returned and post-study interviews conducted, the resulting written, photographic, and audio datasets were transcribed into a format beneficial for use with the qualitative data analysis computer software NVIVO. From the 35 individual probe tasks, we reviewed 76 images, 93 captions, 49 diary entries, and 322 min of interview transcription. To effectively analyse these materials, we employed Braun and Clarke's (2021a) reflexive approach to thematic analysis, which we found offered a level of theoretically flexible as well as the critical reflection necessary to interrogate the characteristics of resolution in imagery of aging. Used widely across the social, behavioral, and applied sciences, this format of thematic analysis has been effective in responding to (a) people's experiences, views or perceptions; (b) understandings or representations; (c) factors or social processes that engage with phenomena; (d) rules or norms that deal with human behavior or practices, as well as this behavior or practices themselves; and (e) the construction of meaning (Braun and Clarke, 2021b). Further, as we seek to engage with methods that support a disciplined practice of critically interrogating what we do—the how and why—the reflexivity central to this approach engages with the role and actions of the designer, valuing their subjectiveness, situatedness, and awareness, questioning them and their work.

Following Braun and Clarke's six phases of thematic analysis, the photovoice, social diary, and semi-structured interview datasets were reviewed individually and then coded by the author to develop themes that spoke to the topics of the study. Encompassing the stages of thematic analysis refined by Braun and Clarke, the author undertook a process of (1) dataset familiarization through a combination of transcription and digitizing of the response, (2) data coding on the NVIVO software then took place; (3) where initial themes were generated and sorted into the overarching topics; (4) these themes were developed and reviewed; (5) which led to further theme refining, defining, and naming; and (6) resulted in the write up of this analysis in a narrated form. Specific to this study, we took a deductive orientation to code the data and sorting themes, with the first exercise in coding for social technology, networks, practices, and wellbeing (Section Social interactivity in later life). This was supplemented by a second exercise sorting themes around the conceptual idea we sought to understand through the dataset—image resolution (Section Perspectives on producing imagery of aging). Pseudonyms have been used for participants, with identifying features such as faces, names, etc., obscured.

### Social interactivity in later life

In analyzing older adults' responses across the topics of social technology, networks, practices, and wellbeing, we documented both technology-dependent and more general forms of interaction, suggesting that social interactivity captures concerns for interpersonal human interaction that we found. While technologies may form a key (and growing) role in social interaction, respondents were more likely to describe its utility or friction in relation to other aspects of their everyday. This point, and other insights, were determined as the author systematically coded an initial fifteen to twenty codes for each topic (*n* = 67), which was reviewed and sorted into thirteen initial sub-themes, developed and refined into three overarching themes. These resulting themes are explained below through a short descriptor supported by responding photography and illustrative quotes.

#### Adapting to a changing social network and personal identity

As foremost a comment on how an individual's social network and personal identity can change over time, the theme of adapting to the shifting, and in many ways reducing, groups an older adult has around them resonated strongly with respondents. As Joan framed it, as a senior, “your social networks do change because people retire and … quite a lot of people are moving into lifestyle communities or retirement villages”. Other respondents also captured this perspective in their own lives, as Mary discussed how an annual camping trip with friends needed to be adapted to a holiday house as the group's interests and accessibility needs changed, with the group itself having dwindled as many passed away. Meanwhile, as a retiree, Claire had tried to join groups with other retirees in their 70s and 80s, which she found “a bit challenging because that's not very stimulating. For me, it's not just trying to find that set of people in my own age group”.

**Figure 4:**
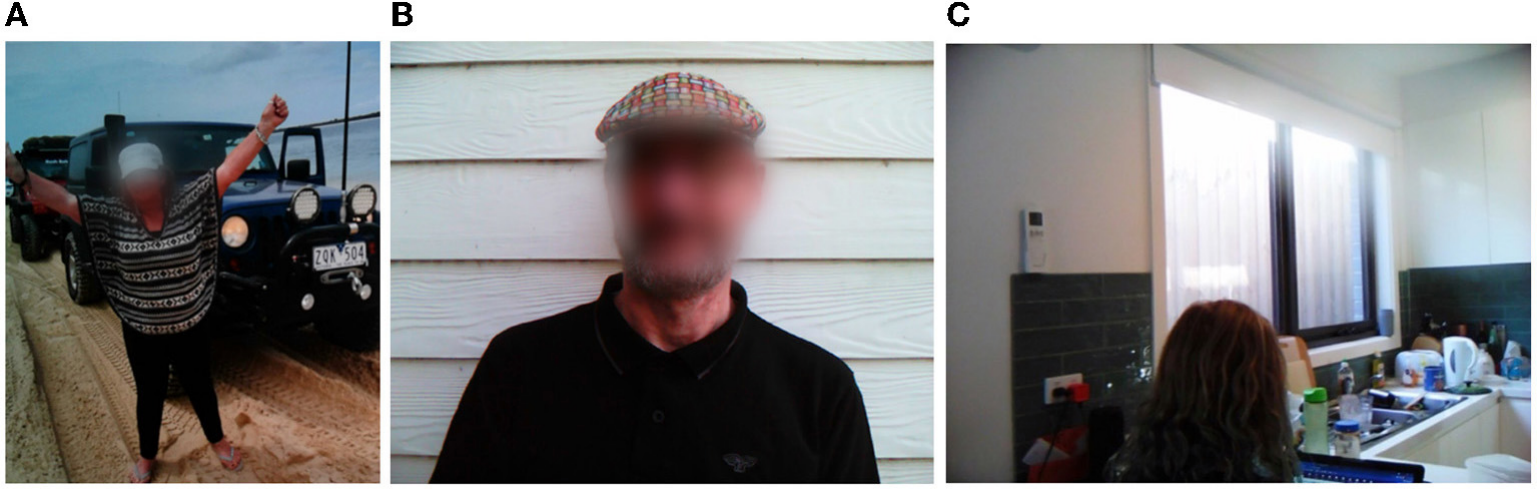
Adapting of social network and identity—**(A)** 4WD club membership, **(B)** wearing your identity, **(C)** helping grandchildren study.

This saw respondents adapting to not only new social groups, but also a changing personal identity, which formed across a spectrum of assimilation to rebellion. For Robert, “You are what you wear—your smile, your opening line, the questions you ask and whether you empathize or better still agree with someone” (Figure 4B). By extension, others also tried to present themselves in a certain way. For example, Mary, a member of a four-wheel driving club, described how she “would like people to see me as capable and adventurous” (Figure 4A). In the context of her family staying with her during the pandemic, Sally would “like to be seen as a helpful person and a caring grandparent” (Figure 4C). In contrast, Emma noted how she made an effort to “represent myself to others as a quirky type who doesn't fit the groove of a 66-year-old”, which could respond to her adapting to living with housemates who are “all young fellas” yet “connect(ing) because we have the same sense of purpose and belonging”.

#### Encountering the social pressures to conform to technology use

**Figure 5:**
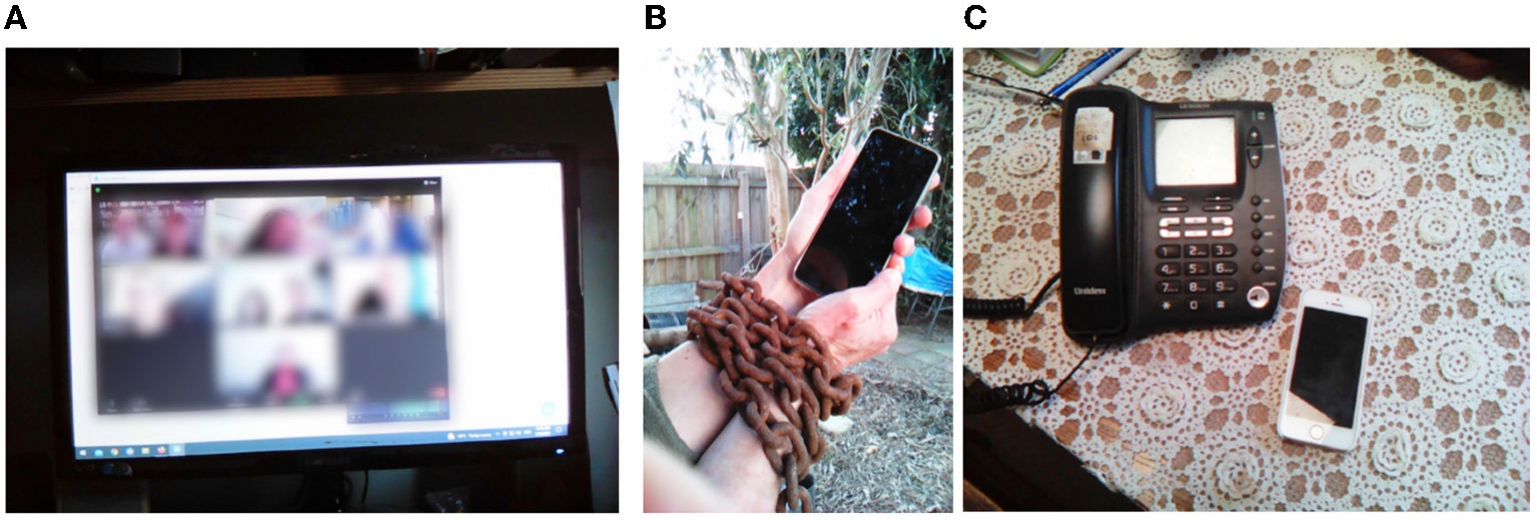
Social pressure with technology—**(A)** zoom participation, **(B)** chained to our devices, **(C)** relying on essential communication technologies.

When discussing their relationship with technology, participants found tensions arose in being social online and the unique pressures to adapt and engage *via* their devices. As Sally summarizes, the emergence of social technologies “means that I can continue my connectedness and my relationships and the groups that I'm involved with, we've all moved online now…” however, this does pose the broader issue for her that “if I didn't have the technology, I'd be out of the loop” (Figure 5A). For most respondents, who demonstrated a medium level of digital literacy, there was a concern with the lack of functionality of social technologies, illustrated by Claire, who relied “on technology or use technology a lot but I'm not really … interested in Facebook. I don't have TicToc, that kind of things not for me, that's time-wasting kind of stuff”.

While this concern about time-wasting was salient, Robert highlighted how there was a “current climate where everybody is supposed to be socially connected”, which he found challenging and provided friction. He communicated this by captioning an image of him holding a phone while wearing chains (Figure 5B), with the question: *Slaves to the tech or slaves to the app or slaves to people's expectations?* This concern of conforming with social pressures or technology requirements framed many interviews, with the increasing dependence on devices to remain connected during lockdowns affecting many (Figure 5C). For example, Sally observed that “I don't like zoom meetings, I find people don't interact in the same way”. While this technology enabled her connectedness and relationship maintenance, she lost out on being able to engage with others in-person in class settings. Meanwhile, Claire co-opted the term “social migraine” to describe how in social situations, she had begun to “realize now after a certain amount of time I want to go—I've actually had enough”, providing tensions to her concerns she was not “social enough” and did not interact with “people enough”. Such a “correct” level of social connection appeared to arrest many respondents, as these pressures meant having to learn and take up social technologies.

#### Being influenced by and reflecting upon the environment

**Figure 6:**
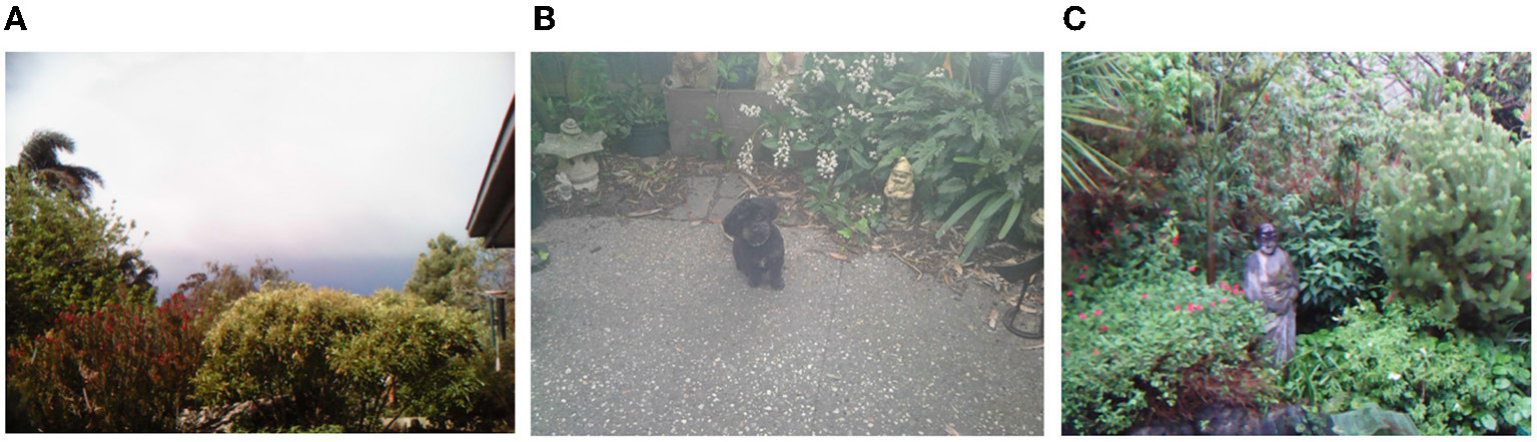
Connecting with nature—**(A)** weather, **(B)** beloved pet, or **(C)** remembering others.

Across the camera activities, there was an emphasis on how aspects of the local environment, such as gardens, pets, or scenery, greatly influenced the wellbeing and mood of individuals while also leading to greater reflections on their lives. For example, responding to the question “how might you feel day to day?”, Emma captured images of the weather, documenting how the “gray days were the fog and ominous clouds hang around drag me down” (Figure 6A) while when “the sun came out and stayed out for the whole day, all is well”. When wellbeing was raised in Joan's interview, she discussed the importance of “just being out there walking or just looking at nature, just being appreciative of how fortunate we are that we live in this country… I feel fortunate. I feel connected to all that as well”.

In the context of the pandemic, animals and gardens offered essential points of connection and reflection for many respondents. Olivia and Mary discussed how spending time with their respective cat family, “Zock Zock” and “Puppy Harli” (Figure 6B), was important to them. At the same time, Emma described being “proud of the beautiful garden I have created”. Gardens formed not only spaces for living but also reflection, as two respondents explained how statues in their gardens represented lost family members, with the monk statue in Emma's Garden bringing her joy in remembering how much the deceased had loved it (Figure 6C). Olivia's statue represents children lost in miscarriage, something she was reflecting on as a close friend's son, his wife, and a 5-week-old baby boy diagnosed with COVID.

### Perspectives on producing imagery of aging

Alongside the topics the probe explored, there was an emphasis on gaining feedback about the respondents' experience of engaging with this cultural study in hopes of better understanding how participants felt portrayed and discussed, reflecting on imagery of later life. This involved analyzing the post-study interviews and a final comments section provided to participants, with the text systematically coded into seventeen basic codes. These were either clustered or propositioned into seven initial themes, which were eventually refined into the following three themes.

#### The imperfect and subjective lens

As an important component, the camera-related activities, and the camera itself, were discussed in depth by participants, who reflected on their approaches to, and motives for, photography, interrogating their own perspectives and decisions in this process. For many, this formed in highlighting their lack of skill or experience behind the camera, with Sally suggesting, “I'm not a great photographer” and Joan “I'm not the world's best with the camera”. Exploring this further, both commented on the type of camera, with a viewfinder in the far corner, which had made aligning an image complex, that the “lens wasn't always exactly where I thought it was going to be”. However, after describing these limitations, Sally noted, “I think it's more about the idea of it, you know, and then the beauty and etc.”, emphasizing a rough and ready nature to the resulting pictures.

This tension between wanting to capture imagery and managing equipment limitations was coupled with a sense of curating and providing the image respondents wanted. For example, Olivia explained that she “really wanted to do more photos and but did stuff up a few and threw them out,” detailing how she became selective and self-editing with these “authentic” pictures. Issues with getting the “right image” persisted for Mary, too, as she struggled with capturing “social technology” because she felt she “didn't know how to take photos of it to be honest”. Nevertheless, this transparency around the limitations of the camera and their perceptions of “good images” indicated how the camera activities provided an invaluable resource for recording images of aging: this and other modes needed to be used with an understanding of their pitfalls and complications.

#### Self-reflections and the portrayal of older adults

This approach to engaging with lived experience saw many respondents indicate how they had reflected on or realized something important about themselves. This was apparent in how Olivia discussed having not “appreciated how well supported I am” as a housebound individual. The diary component elicited that she had “realized that I do actually have a routine in the mornings”. For Robert, this involved considering how he “only has a couple of friends, but a number of acquaintances”, reflecting on his social practices as someone who finds that “interactions with acquaintances or random people are often difficult to navigate”.

Robert had also considered how the activities might portray him, forming concerns about being defined by the scope of the probe study as an individual. Because the emphasis of the study was on the domains of social technology, network, practices, as well as wellbeing, he felt the study was “very oriented toward how many connections and family and friends that you have, and I have bugger all, I have less now than I ever did have”. In being transparent in his final comments, he explained how this had formed “a suspicion that I will be portrayed in the study as a friendless, lonely, and consequently unhappy person, who isn't coping with life, which isn't the case”. This discussion provides an essential understanding of how our activities and work can lead to certain portrayals of individuals and broader assumptions of aging. While cultural probes are often advocated for due to their sensitivity to people's lives and signifying reciprocity (Çerçi et al., 2021), this does not always happen, and the need for transparency became paramount with these findings.

#### The qualities and limitations of cultural probes

First, as foregrounded, cultural probes have been perceived to enable a level of autonomy and authenticity that other methods can lack; however, while they can provide challenging or thought-provoking activities, not everything can connect or sit well with respondents. Across many of the final comments attached to the study, respondents described how the activities were uniquely engaging, with Emma seeing it as “thought-provoking and some of them are quite confrontational”, while Mary also summarized how she found the “whole project interesting, sometimes challenging, but thought-provoking.”

While this provided evidence of how a cultural probe study can reconfigure the creative and participatory research experience, this method does have some notable limitations for many respondents. For example, Claire found that “some questions felt like they were probably for someone older than me”, suggesting the study likely had a wide age range and that she was “young to be retired”. Specifically, regarding the questions posed, both Emma and Robert noted issues with the Social Identity activity, with Emma indicating it “confused me a bit and I didn't know if I filled it in correctly or if it was worded that way to explore our perceptions of exactly what a ‘social identity’ is”. Meanwhile, Robert found the “Social and Personal Identity Charts have practically no meaning for me and are quite alien in concept. I don't relate my identity to them in any meaningful way”. This feedback helps shape future research and encourages more discussion around how older adults perceive social and personal identity to find new ways to communicate and explore these topics.

## Discussion

Having framed this article around the issues and inconsistent efforts to evaluate imagery capture and processing used by designers, we have sought to examine whether being able to characterize the resolution of images formed by designers can provide “better” imagery of later life. In considering the question of “what characterizes ‘better’ images of aging created by designers?” and Fischer et al.' emphasis on designer's reflective practices in configuring participatory design, we also conclude that the actions, choices, and agendas of designers shape their portrayal of later life. Our study illustrates how specific approaches to developing participant materials, from the chosen quantitative scales to the photovoice prompts, can not only direct the focus of imagery but also produce incomplete or misrepresentative pictures. Rather than determining what images are correct or “good”, those that produce imagery of older adults (from designers to researchers to the media and the public) might form “better” images by offering more transparency in our processes of production, alongside having increased reflectivity on the methods of creation and analysis chosen. As the feedback and discussion with participants demonstrates, it can be helpful to examine the input resolution of chosen activities toward determining what limitations or issues may arise, as well as interrogating how the configuration of output methods can enable or diminish participant voices. In this discussion, we focus on reflecting on our hypothesis of “resolution”, forming a concept that can support designers in forming better imagery of later life.

As hypothesized, the cultural probe study demonstrates how input resolution can characterize the vehicle used to collect sources of information toward forming an image, that this input can be viewed as a spectrum of image production processes. This spectrum, we suggested, saw cultural probe studies provide higher resolution to what is captured than less direct and perhaps more stigmatizing and biased second-hand imagery. Responding to Kathrina Dankl's assertation that “vision, multidimensional inquiry and implementation *via* engagement” (p. 39) are essential to resisting persistent imagery of later life based on models of deficiency, this spectrum would serve in valuing the “human experience of aging” and “culturally informed insights” (p. 38). In reflecting on how the method employed here provides more resolution, we document how participation not only in the research but by an independent project advisor added layers of accessibility and intuition to the activities that others have also seen improve resulting imagery (Mikus et al., 2022). This input enabled the study to capture relevant insights and provide a challenging and thought-provoking experience for participants. The level of feedback from participants was also instrumental in realizing the limitations of input resolution for photovoice tasks, highlighting how a process that places a camera into the subject's hands needs to consider the implicit imperfectness and subjectiveness of the resulting media. In recognizing these nuances and considering them alongside the resulting imagery, utilizing primary source methods and high levels of participation can see higher input resolution produced.

In considering the degree of output resolution—the processing and analysis of captured imagery—there is a clear benefit to incorporating reflexive processes that involve researchers and participants. Evidenced in the self-reflections and further concerns around how they are being portrayed, participants can respond to the research materials themselves and be critical of the implications and implicit directions such studies can take. Design researchers Li et al. (2020) support this type of empathic design in developing products, as in the case of health monitoring wearables. Multiple perspectives, as well as concern for semantics and ethics, are invaluable in addressing the stigmas and perceptions of older adults. In light of this, output resolution can provide a more explicit connection between preconceptions, imagery, and the design context. Higher resolution forms images of aging that are better because they detail caveats and offer a reflective depth. Adding these nuances to the future images of aging formed by designers can realize the limitations and discuss potential biases, helping to reduce stigmatization, such as an over-emphasis on cognitive and physical aging (Dankl, 2017). In this way, both input and output resolution can provide a criterion for relevance and strength of such interventions.

Using a cultural probe study to interrogate and characterize images of aging through the methods described here offers benefits and limitations. As presented, like many cultural probe studies, we found it feasible to only conduct the probe with a small number of respondents, which means that the findings are not representative of a large population, however, such scale is not the purpose of the cultural probes methodology (Celikoglu et al., 2017). In addition, the reflexive thematic analysis described here offers an invaluable level of flexibility as well as provides powerful and persuasive discourse. However, we highlight that this reflexive does celebrate the researcher's reflexivity and intersubjectivity, making the methodological integrity of the process critical to enabling trustworthiness in our work (Finlay, 2021). Finally, reflecting on the values this research places on designers examining their practices, this analysis format can also support them to take more reflective and thoughtful engagement with their approaches.

## Conclusion

As designers encounter inconsistent and often erroneous representations of older adults in their work, we have sought to provide critical tools to help characterize what better images of aging can be. The qualities of resolution, recognizing the role of input and output processes, can offer designers a way to characterize their resulting images of aging, utilizing ones that provide pathways to understanding and including older adults in their work. The nuances discussed here aid in determining the degree of input and output resolution, indicating the potential for resolution as a characteristic that can be used to detail and explain the images of aging we form. While this cultural probe study explored issues of technology use and social isolation during the COVID-19 pandemic, we also sought to capture participants' perspectives on the study itself toward better realizing the benefits and limitations of this method. In considering the resolution of imagery produced throughout their work, designers should be able to ascertain what “hard facts” they have unearthed while articulating the fidelity of these representations of later life. We call for this conceptualization of resolution to be considered and tested by designers in their future image production. We also consider how these high-resolution images could be represented or integrated into design projects outside the co-constitution of aging and technology across the design field more broadly. Through engaging in participatory and reflexive modes of generation and analysis, we ask that designers seek to realize better images of aging rather than engaging with stigmas and ignoring the ethical ramifications of their work.

## Data availability statement

The raw data supporting the conclusions of this article will be made available by the authors, without undue reservation.

## Ethics statement

This case study involving human participants was reviewed and approved by RMIT University Design and Social Context College Human Ethics Advisory Network (DSC CHEAN). The patients/participants provided their written informed consent to participate in this study. Written informed consent was obtained from the individual(s) for the publication of any potentially identifiable images or data included in this article.

## Author contributions

JS provided a substantial contribution to the article by conducting the study, forming the concept that drove its development, and documenting these outcomes as part of this piece.

## Conflict of interest

The author declares that the research was conducted in the absence of any commercial or financial relationships that could be construed as a potential conflict of interest.

## Publisher's note

All claims expressed in this article are solely those of the authors and do not necessarily represent those of their affiliated organizations, or those of the publisher, the editors and the reviewers. Any product that may be evaluated in this article, or claim that may be made by its manufacturer, is not guaranteed or endorsed by the publisher.
